# mGem: Tapping into the language of symbiosis to advance human microbiome research

**DOI:** 10.1128/mbio.03661-25

**Published:** 2026-06-09

**Authors:** Gina R. Lewin, Lily Khadempour

**Affiliations:** 1 Center for Global Health and Diseases, Department of Pathology, Case Western Reserve University School of Medicine 12304, Cleveland, Ohio, USA; 2 Case Western Reserve University-Cleveland VA Medical Center for Antimicrobial Resistance and Epidemiology 2546, Cleveland, Ohio, USA; 3 Department of Earth and Environmental Sciences, Rutgers University, Newark 67206 , Newark, New Jersey, USA; Georgia Institute of Technology, Atlanta, Georgia, USA

**Keywords:** commensal, mutualist, parasite, pathogen, microbe-host interactions, symbiont

## Abstract

In human microbiome research, the term “commensal” is often used to describe organisms that benefit their hosts. In ecology, in host-microbe symbiosis, a commensal organism has no impact on its host, whereas a mutualist organism benefits its host. While others have recognized this discrepancy in terminology use, old habits are hard to break, and the human microbiome community has continued in this vein. This is our call to action for the human microbiome community to use more precise terminology that appropriately reflects the impact that these microbes have on their hosts. We should use the terms “commensal” and “mutualist” when we know the effect on the host, and “symbiont” when we do not. By using the same terminology as ecologists, we will be able to make use of, and contribute to the vast research in the field of symbiosis.

## PERSPECTIVE

An overarching goal in microbiome research is to understand how the complex communities in our bodies contribute to health and disease, and ultimately, we aim to leverage and control these communities to promote health. To continue advancing this work, we need conceptual frameworks that aid in understanding the relationships between us and our microbiota. Conceptual frameworks can ensure that we avoid doing our science in silos by unifying work across disciplines, and they can help form hypotheses and questions for future research. In human microbiome research, there have been various conceptual frameworks that have advanced our science (1–4). In this perspective, we highlight the symbiosis framework and definitions in connection to microbiome research, with the hope that adoption of this framework will advance our ability to study the human microbiome.

**Fig 1:**
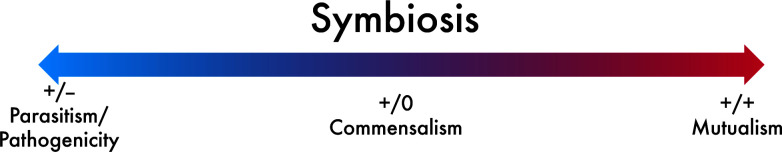
Symbiosis exists on a spectrum where relationships can shift over time and space. For host-microbe interactions, we assume that the microbe benefits across all relationships, but the impact on the host can range from harmful (parasitism) to neutral (commensalism) to beneficial (mutualism).

The symbiosis framework helps us to understand and describe the relationship between human hosts and their microbiota (5). Symbiotic relationships exist on a spectrum between mutualism, where both partners benefit, to parasitism (or pathogenesis), where one partner is harmed while the other benefits. Between these two extremes is commensalism, where one partner experiences no effect while the other benefits. Importantly, these are not discrete categories but exist along a spectrum, and the location along the spectrum can change depending on context (Fig. 1). In host-microbe symbioses, it is assumed that the microbe always benefits in the symbiosis while the impact on the host varies (5). Thus, the relationship between humans and members of their microbiota can range from beneficial to harmful, and the outcomes of the relationship can change depending on context (immune status, other microbes that are present, diet, drugs, etc.) on both ecological and evolutionary scales.

Below, we show examples of how both in classic ecological literature and in human microbiomes, we can observe shifts across this axis.

In the ecology and evolution literature, there are countless examples of transitions along the axis of fitness effects (see the excellent review by reference 6). Within these examples, there are cases where the shifts can be drastic: Cicadas have repeatedly recruited a fungal mutualist that provides essential amino acids to its host, but these fungi originate from a parasitic clade, *Ophiocordyceps*, that are typically responsible for insect “zombie” infections (7). Jellyfish and corals (cnidarians) rely on Symbiodiniaceae algae as classic mutualists that provide photosynthates to their hosts, but can become parasitic when the host experiences stress, usually due to extreme temperatures (8, 9). In other cases, the transitions can be more subtle. For example, myxoma viruses were introduced to help control invasive rabbit populations in Australia, but the virus evolved to be less pathogenic, and the rabbits evolved increased resistance. Both evolutionary shifts moved the relationship along the spectrum from extremely pathogenic/parasitic to less pathogenic/parasitic (10).

In the human microbiome literature, we can see similar context-dependent shifts along the axis of fitness effects. The most common examples are opportunistic pathogens, organisms that are typically commensal or even mutualistic but that become pathogens when the host is immunocompromised or if the microbe ends up in the wrong part of the body. For example, *Candida albicans* is commonly present in the human oral cavity, gut, skin, and vagina, without causing disease. However, under some circumstances, *C. albicans* can become pathogenic within those sites, for instance, causing oral or vaginal candidiasis, or even causing systemic infection (11). Similarly, *Staphylococcus aureus* can colonize the nose and other sites within the human body without causing disease, and in this state, it may provide benefits to the host, including protection against other pathogens through trained immunity (12). Yet, within certain hosts or environments (e.g., chronic wounds or joint infections), *S. aureus* is able to upregulate virulence factors and promote disease (13). Many members of the microbiota have been reported to cause disease in rare cases. For instance, *Rothia* species are abundant in the oral cavity and upper respiratory tract, where they are thought to behave as mutualists through respiring nitrate and inhibiting pathogens, but in a small number of cases, *Rothia* has been reported to cause infections such as bacteremia, endocarditis, and pneumonia (14). Finally, over evolutionary time, pathogens can shift in virulence, and it is contested if there are underlying rules or patterns for these changes (15, 16). In many cases, pathogens decrease in virulence over time, as in the case of *Yersinia pestis*, where a decrease in the genomic copy number of a protease virulence factor may have led to decreased mortality towards the end of the first and second plagues (17). In other cases, pathogens have been thought to evolve from less pathogenic ancestors, such as in the case of *Mycobacterium tuberculosis* (18).

There are many different terms that are used to acknowledge the complexity and fluidity of the relationship between humans and members of the microbiota, such as opportunistic pathogen, pathobiont (19), or commensal. Human microbiome researchers routinely refer to any member of the microbiota that does not cause harm as a commensal microbe, encompassing both mutualists and actual commensal microbes, which is imprecise. In addition, if the researchers do not know what effect the microbe has on the host, commensal is often used as a catch-all. This convention arises largely from historical usage. For over 100 years, scientists have recognized that our bodies are covered with bacteria, including our intestinal flora and other potentially pathogenic microbes that are not actively causing disease (20). While Pasteur postulated that microbes were essential for animal survival in 1885 (21), it was not generally accepted that these microbes were beneficial. Instead, over time, they were termed commensal (22), and that terminology has continued, even as our understanding of the human microbiota has expanded to further understand the role of these microbes. Thus, while many modern researchers recognize that the term commensal does not accurately describe members of the human microbiota, they use it as a convenient term.

We recommend that, instead of commensal, the term symbiont be used as a general term to describe a member of the human microbiota until more is known about its relationship with the host. Because symbiont does not imply benefits or harms, it also encompasses the nuances of terms like opportunistic pathogen or pathobiont. This shift in terminology is helpful for advancing the human microbiome field. One simple reason is that it is important to be able to differentiate between mutualists and commensals, since they are not the same thing. We should all aspire to use the most accurate terminology when describing our science. Of course, it is not just about terminology, but about understanding the commonalities and differences between pathogenic, commensal, and mutualistic microbes. These host-associated, or symbiotic, microbes all require adaptations that allow them to survive in association with a host. One idea that has been proposed is that in order for any microbe to be a symbiont, it first must be adapted to living with a host, regardless of where the relationship exists on the spectrum of fitness effects from mutualism to parasitism (18, 23). Taking this perspective, that first comes symbiosis then comes the particular fitness effects on the host, emphasizes the importance of understanding the underlying host-microbe interactions, no matter where the interaction falls along the symbiosis spectrum.

Understanding the factors that mediate interactions, and ultimately what causes an organism to move along the spectrum towards mutualism or pathogenicity, is a major focus of the symbiosis field, but is also important for identifying therapeutic approaches for improving human health. For example, in the cnidarian-Symbiodiniaceae symbioses, researchers are working to understand the triggers that cause the symbionts to move from being mutualistic to parasitic. It appears that the Symbiodiniaceae increase their production of reactive oxygen species as they shift from being mutualists to parasites of their host (24). Similarly, in the human microbiome, ornithine has been shown as an important metabolite for shifting organisms towards pathogenesis, including in the gut (25) and urinary tract (26). The switch from mutualist to pathogen is not always symbiont-mediated, however. *Bifidobacterium*, a genus of bacteria that are only typically thought of as beneficial in humans, can also cause disease under the right circumstances (27). In this study, Esaiassen et al. found that there were no obvious pathogenicity traits found in the disease-causing *Bifidobacterium* strains, but rather the disease was caused largely due to the susceptibility of the host.

We recognize that the term commensal, as commonly used in the microbiome field, is inaccurate and limits how we envision the role of the microbiota. By moving to call these microbes symbionts, it emphasizes the complexity of our relationships with these organisms. Many abundant human symbionts are not well studied (28), and there is much to learn about how to promote human health through a better understanding of their roles. In addition to an axis of fitness effects, which we explore here, there are additional axes that can help to define aspects of symbiosis (partner fidelity, transmission mode, and level of intimacy) that can further contribute to human microbiome science (5). There is rich knowledge in other fields that study host-symbiont interactions, and it is helpful to use the same terminology so we can make use of that knowledge and contribute to it. Our call to action is to ask our colleagues in the human microbiome research fields to revise their terminology and to adopt the symbiosis framework.
